# ASGR1 - a new target for lowering non-HDL cholesterol

**DOI:** 10.21542/gcsp.2016.14

**Published:** 2016-06-30

**Authors:** Mohamed Hassan, Kerolos Wagdy

**Affiliations:** Division of Cardiology, Aswan Heart Center, Aswan, Egypt

## Abstract

Non-HDL cholesterol (non-HDL-C) has been introduced as an alternative cardiovascular (CV) risk marker and a secondary therapeutic target in patients with combined hyperlipidemia, diabetes mellitus, metabolic syndrome, or chronic kidney disease. An important genetic study on the Icelandic population has recently identified a strong link between a new gene – ASGR1 (for asialoglycoprotein receptor) – mutation, plasma non-HDL-C levels, and coronary heart disease (CHD). Heterozygous carriers of a rare noncoding 12-base-pair (bp) deletion (del12 deletion) in intron 4 of ASGR1 had a 13.6 mg/dl lower level of non-HDL-C and a 34% lower risk of CHD than non carriers. The cardioprotective effect of ASGR1 loss-of-function is surprisingly larger than predicted by its effect on the levels of non-HDL-C, which suggests that the atheroprotective effects of del12 mutation go beyond the lowering of serum cholesterol levels. This has shed some light on a new path – the sialylation pathway – possibly leading to a novel therapy that neutralize ASGR1 for heart disease prevention and treatment.

## Introduction

Assessment of non–high-density lipoprotein cholesterol (non-HDL-C) provides a measure of cholesterol contained in all atherogenic apo-B containing lipoproteins (low density lipoprotein “LDL”, very low density lipoprotein, intermediate density lipoprotein, lipoprotein (a), chylomicrons, and chylomicrons remnants). Non-HDL-C has been shown to be a better predictor of cardiovascular (CV) risk than LDL cholesterol (LDL-C).^1^ A large meta-analysis demonstrated a 1:1 relationship between percent non-HDL-C lowering and coronary heart disease (CHD) reduction.^2^ Moreover, in the EPIC-Norfolk prospective population study, high non-HDL-C levels was associated with increased risk for CHD independent of LDL-C levels in participants without diabetes or CHD, followed up for 11 years.^3^ Non-HDL-C has been suggested as an alternative CV risk marker and a secondary therapeutic target in patients with combined hyperlipidemia, diabetes mellitus, metabolic syndrome, or chronic kidney disease in the third Adult Treatment Panel (ATP III) guidelines of the US National Cholesterol Education Program,^4^ and latest European Society of Cardiology (ESC) guidelines.^5^ The non-HDL-C target has been reported to be less than 100 mg/dL, and 130 mg/dL in those at very high, and high total CV risk, respectively (class IIa recommendations, level of evidence B).^5^ However, no recommendations for, or against, specific non-HDL-C target have been made in the latest American College of Cardiology (ACC) guidelines.^6^

**Figure 1.**
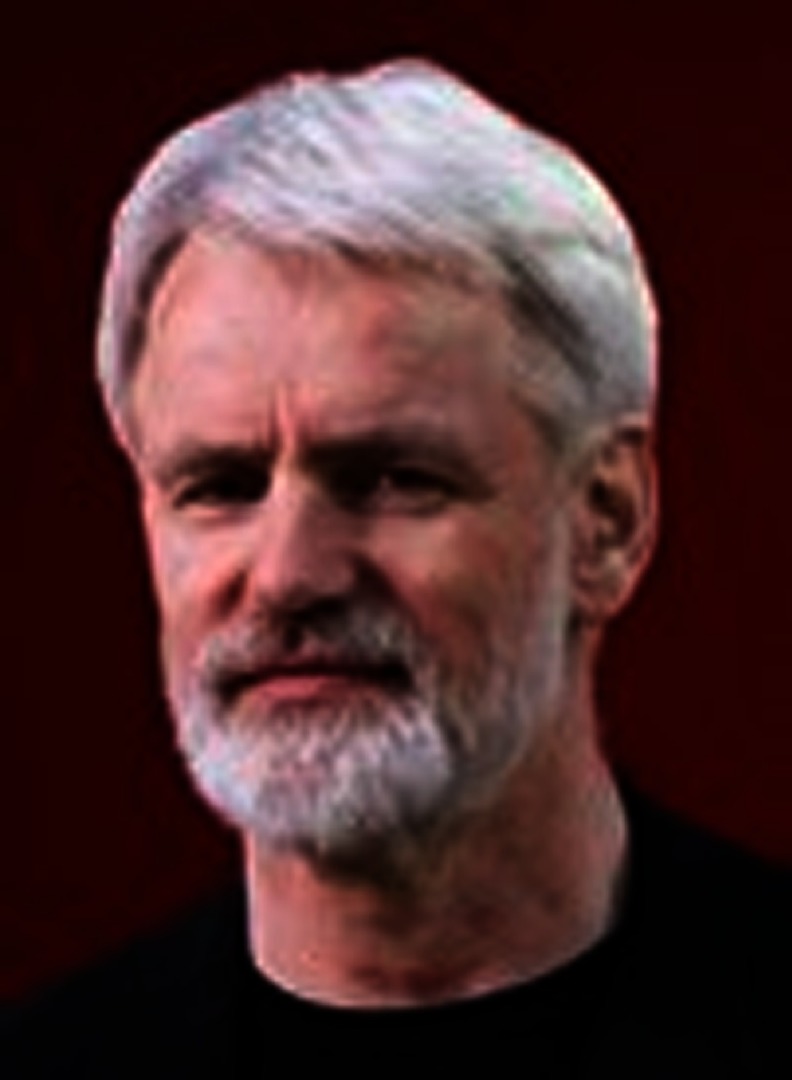
Kari Stefansson.

Translational research has identified several gene mutations (for example: proprotein convertase subtilisin kexin 9 “PCSK9”, Apoprotein C3 “APOC3”, Angiopoietin-like 4 “ANGPTL4”, and Niemann Pick C1-like protein 1 “NPC1L1” genes) that were associated with significant favorable changes in plasma levels of non-HDL-C and subsequently reduction in CHD risk.^7–15^ Currently, researchers still exert tremendous efforts to translate these findings into clinical applications in order to discover novel therapeutic targets that can reduce the burden of CV diseases An important genetic study by Dr Paul Nioi, based on the Icelandic population, has recently identified a strong link between a rare loss of function (LOF) mutation in the gene of asialoglycoprotein receptor 1 ( ASGR1), non-HDL-C, and CHD.^16,17^ The ASGR1 gene was initially identified by Kari Stefansson -a leading figure in human genetics (Figure 1). The ASGR1 gene encodes the major subunit of asialoglycoprotein receptors (ASGPR). The latter family was initially identified and characterized by Ashwell and co-workers,^18^ as type-II transmembrane proteins that play a critical role in serum glycoprotein homeostasis by mediating the endocytosis and lysosomal degradation of asialoglycoproteins (desialylated glycoproteins; glycoproteins from which a sialic acid has been removed to expose galactose or N-acetylgalactosamine residues). These receptors are highly expressed on the surface of hepatocytes and several human carcinoma cell lines.^19^

## ASGR1 genetic variants, non-HDL-C, and CHD

**Figure 2.**
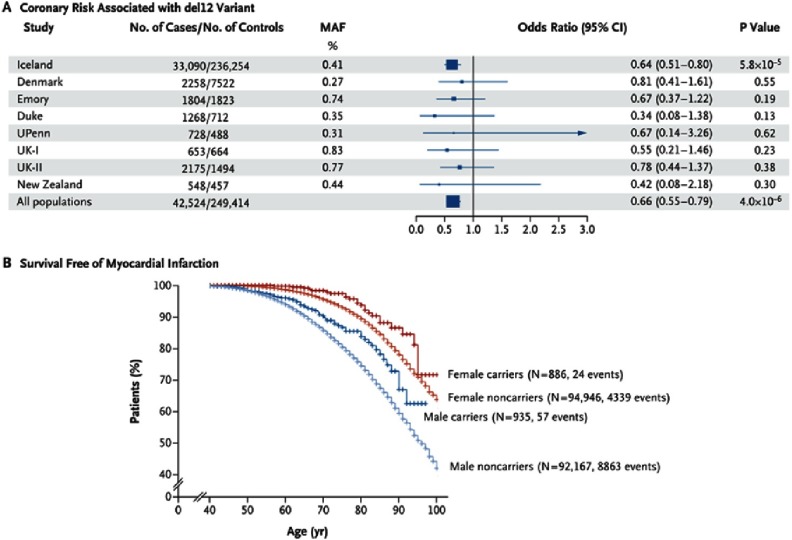
Association of del12 in ASGR1 with a reduced risk of coronary artery disease and myocardial infarction. *Panel A:* odds ratios for coronary artery disease associated with the del12 variant in ASGR1 among a total of 42,524 participants with coronary artery disease and 249,414 controls in Iceland, Denmark, the United States (Emory University, Duke University, and the University of Pennsylvania [UPenn]), the United Kingdom (UK-I and UK-II), and New Zealand. MAF denotes minor allele frequency. *Panel B*: shows Kaplan–Meier curves for survival free of a first myocardial infarction among heterozygous carriers and noncarriers of the del12 variant, stratified according to sex. Reproduced with permission from *New England Journal of Medicine*.

The study, accompanied by an editorial written by Dr Anne Tybjærg-Hansen (University of Copenhagen, Denmark), has been recently published in the *New England Journal of Medicine* in May 2016.^16,17^ The genomes of 2,636 Icelanders were sequenced and 25.3 million sequence variants have been identified. Those variants have been imputed into the genomes of additional 398,000 living and deceased Icelanders. Among them, 119,146 had information on serum non-HDL–C levels. The possible effect of LOF *ASGR1* variants on the risk of CHD has then been explored in 42,524 cases and 249,414 controls from five European ancestry populations (Figure 2).

A rare noncoding 12-base-pair deletion (del12) in intron 4 of ASGR1 has been demonstrated in 0.8% of the study population. The deletion activates a cryptic splice site that leads to frameshift mutation and a shorter protein that is prone to rapid degradation, thus is considered as a LOF allele. Heterozygous carriers of del12 mutation of ASGR1 had a 13.6 mg/dl lower level of non-HDL-C than non-carriers (relative risk reduction 9%, P = 1.0 × 10^−16^), and a 34% lower risk of CHD (95% confidence interval “CI”: 21–45; P = 4.0 × 10^−6^). This reduction included a 9.5 mg/dl decrease in LDL-C levels (relative risk reduction 7%) and a 6.1% reduction in triglycerides levels. This was associated with nearly 50% increase in plasma levels of alkaline phosphatase, and a smaller increase in vitamin B_12_ (cobalamine) levels. On the other hand, a second rare variant ASGR1 W158X (found in 1 in 1,850 persons) has been also identified by the group and was associated with even more pronounced reductions in non-HDL-C, LDL-C, and triglycerides levels.

This favorable reduction in CHD risk has been demonstrated in both the Icelandic set (P = 5.8 × 10^−5^) and the non-Icelandic sets (P = 0.02). The study reported a combined odds ratio of 0.66 (P = 4.0 × 10^−6^) (Figure 2), and did not show any evidence of heterogeneity across the eight study populations (P = 0.96).

## Discussion

*ASGR1* variants have been identified to have a significant favorable impact on lipid levels and CHD. ASGR1 variants had a larger effect on CV risk than is predicted by its effect on levels of non-HDL-C, which suggests that the atheroprotective effects of ASGR1 del12 go beyond the lowering of serum cholesterol levels. The precise mechanism of such effect on plasma lipoproteins and subsequent impressive CHD risk reduction remains largely unknown. It could be partially explained by enhanced apolipoprotein B–containing lipoprotein clearance. ASGPR has been implicated in the metabolism of LDL particles, chylomicron remnants, and lipoprotein (a).^20,21^ It may contribute to the endocytosis of chylomicron remnants mainly, and to lesser extent the LDL particles.^20^ ASGPR may interact with the asialylated form of the LDL receptor promoting its recycling through endocytosis by the plasma membrane of hepatocytes, thereby affecting LDL-C levels.^22^ Moreover, the catabolism of asialo-Lp(a) in ASGPR-mice was 8-fold faster than native Lp(a) in wild-type mice.^21^ On the other hand, it seems that there are other cardioprotective pathways affected by this gene. For instance, sialylation of chemokines or their receptors has been reported to influence the recruitment of inflammatory cells to atherosclerotic plaques.^23,24^

ASGR1 mutations may have a strong impact on CHD risk, but not as large as for PCSK9 or APOC3.^7,16,25,26^ PCSK9 partial LOF mutations (e.g. R46L) have been associated with 30% reduction in CHD risk in three independent studies and meta-analyses (odds ratio 0.70, 95% CI: 0.58–0.86, p < 0.001).^25^ Moreover, complete LOF mutations of PCSK9 (as is the case of ASGR1 del12 or W158X mutations) result in 88% reduction in CV events over 15 years.^26^ On the other hand carriers of APOC3 LOF mutation had 40% lower risk of CHD than that in non-carriers (odds ratio 0.60; 95% CI: 0.47 to 0.75; p = 4 × 10^−6^).^7^

Increased levels of plasma alkaline phosphatase and vitamin B_12_ in the study may be attributed to reduced clearance of such molecules by ASGPR. The isoform of alkaline phosphatase that is derived from the intestine, and the vitamin B_12_ transporter (haptocorrin) are asialylated glycoproteins that has been reported to bind to ASGPR.^27,28^ However, the plausible effects of the ∼50% higher levels of alkaline phosphatase on bone integrity or turnover should be a concern, especially in aged individuals.

## What have we learned?

ASGR1 represent another success story of large-scale genetics. ASGR1 LOF variants had powerful cardioprotective effect which caused impressive reduction in cardiovascular events. This has shed some light on a new path – the sialylation pathway – which could represent an initial step towards a novel therapy that neutralizes ASGR1 for heart disease prevention and treatment. The mechanisms of such favorable effects remain to be determined.
